# Stimulation of cannabinoid receptor 2 (CB_2_) suppresses microglial activation

**DOI:** 10.1186/1742-2094-2-29

**Published:** 2005-12-12

**Authors:** Jared Ehrhart, Demian Obregon, Takashi Mori, Huayan Hou, Nan Sun, Yun Bai, Thomas Klein, Francisco Fernandez, Jun Tan, R Douglas Shytle

**Affiliations:** 1 Neuroimmunlogy Laboratory, Silver Child Development Center, Department of Psychiatry and Behavioral Medicine, University of South Florida College of Medicine, Tampa, FL 33613, USA; 2 Institute of Medical Science, Saitama Medical School, Saitama 350-8550, Japan; 3 Department of Molecular Genetics, the Third Medical University, Chongqing, China; 4 Department of Medical Microbiology and Immunology, University of South Florida College of Medicine, Tampa, FL 33613, USA; 5 Center for Excellence in Aging and Brain Repair, Department of Neurosurgery, University of South Florida College of Medicine, Tampa, FL 33613, USA; 6 Department of Pharmacology and Therapeutics, University of South Florida College of Medicine, Tampa, FL 33613, USA

## Abstract

**Background:**

Activated microglial cells have been implicated in a number of neurodegenerative disorders, including Alzheimer's disease (AD), multiple sclerosis (MS), and HIV dementia. It is well known that inflammatory mediators such as nitric oxide (NO), cytokines, and chemokines play an important role in microglial cell-associated neuron cell damage. Our previous studies have shown that CD40 signaling is involved in pathological activation of microglial cells. Many data reveal that cannabinoids mediate suppression of inflammation *in vitro *and *in vivo *through stimulation of cannabinoid receptor 2 (CB_2_).

**Methods:**

In this study, we investigated the effects of a cannabinoid agonist on CD40 expression and function by cultured microglial cells activated by IFN-γ using RT-PCR, Western immunoblotting, flow cytometry, and anti-CB_2 _small interfering RNA (siRNA) analyses. Furthermore, we examined if the stimulation of CB_2 _could modulate the capacity of microglial cells to phagocytise Aβ_1–42 _peptide using a phagocytosis assay.

**Results:**

We found that the selective stimulation of cannabinoid receptor CB_2 _by JWH-015 suppressed IFN-γ-induced CD40 expression. In addition, this CB_2 _agonist markedly inhibited IFN-γ-induced phosphorylation of JAK/STAT1. Further, this stimulation was also able to suppress microglial TNF-α and nitric oxide production induced either by IFN-γ or Aβ peptide challenge in the presence of CD40 ligation. Finally, we showed that CB_2 _activation by JWH-015 markedly attenuated CD40-mediated inhibition of microglial phagocytosis of Aβ_1–42 _peptide. Taken together, these results provide mechanistic insight into beneficial effects provided by cannabinoid receptor CB_2 _modulation in neurodegenerative diseases, particularly AD.

## Background

Most neurodegenerative diseases are associated with chronic inflammation resulting from the activation of brain mononuclear phagocyte cells, called microglial cells[1]. Because increased proliferation of microglial cells is seen in brains of patients with multiple sclerosis (MS) [2], Alzheimer's disease (AD)[3], and HIV [4]; and because sustained microglial activation, associated with these diseases, is known to have deleterious effects on the surrounding neurons. [5], factors mediating microglial activation are of intense interest.

Marijuana and its active constituent, {Delta}9-tetrahydrocannabinol (THC), suppress cell-mediated immune responses (for review, see. [6]). Many of these effects are mediated by the cannabinoid receptor 2 (CB_2_), as demonstrated by the finding that THC inhibits helper T-cell activation by normal, but not CB_2 _knockout-derived, macrophages [7]. While many studies have investigated effects of cannabinoids on immune function, few studies have examined their effects on the CD40 pathway [8].

The CD40 receptor is a 50 kDa type-I phosphoprotein member of the tumor necrosis factor (TNF)-receptor (TNFR) superfamily, which is expressed by a wide variety of cells [8]. The ligand for CD40 (CD154, i.e. CD40L) is mainly expressed by activated CD4+ T-cells. Following ligation of CD40, numerous cell-type-dependent signaling pathways are activated, leading to changes in gene expression and function. These changes include several signal transduction pathways: nuclear factor kappa-B (NF-κB), mitogen-activated protein (MAP) kinases, TNFR-associated factor proteins, phosphatidylinositol-3 kinase (PI3K), and the Janus kinase (JAK)/signal transducer and activator of transcription 1 (STAT1) pathway. [9,10]. Ligation of CD40 on microglial cells leads to the production of TNF-α and other unidentified neurotoxins [11-13]. Thus, signaling through CD40 on microglial cells induces soluble mediators that could have important functional roles in the central nervous system (CNS).

In the normal brain, microglial cells display a quiescent phenotype, including low CD40 expression [14]. However, upon insult to the brain, microglial cells become highly activated, altering their phagocytic and antigen-presentation functions [15] as well as the production of cytokines [13]. Mounting evidence implicates microglial CD40 as contributing to the initiation and/or progression of several neurodegenerative diseases [15]. In fact, blocking CD40-CD154 interactions by a neutralizing antibody strategy prevents murine experimental autoimmune encephalomyelitis (EAE) disease activity [16-19] as well as AD-like pathology in mouse models of the disease [20].

Given the recently described immunomodulatory role of cannabinoids, the importance of CD40-CD40L interaction in neuroinflammatory diseases, and the clinical and basic science studies suggesting that cannabinoids may be therapeutic in AD and MS, [21-25], we examined, in the present study, whether cannabinoids (primarily CB_2 _agonist JWH-015) could oppose microglial CD40 expression following interferon-γ (IFN-γ) challenge. Furthermore, we examined whether CB_2 _agonist JWH-015 influences microglial phagocytic function and/or proinflammatory cytokine production after CD40 ligation.

## Materials and methods

### Peptides and drugs

Aβ_1–42 _peptide, purity greater than 95% according to manufacturer's HPLC analysis, was obtained from QCB (Hopkinton, MA). Aβ_1–42 _peptide used for all experiments was made fibrillar/aggregated, as previously described [26]. Briefly, 2 mg of Aβ_1–42 _was added to 0.9 ml of pure water (Sigma), the mixture was vortexed, and 100 μl of 10 × PBS (1 × PBS contains 0.15 M NaCl, 0.01 M sodium phosphate, pH 7.5) was added and the solution was incubated at 37°C for 24 hr. The Cy3-Aβ peptide's conjugation was carried out in strict accordance with the manufacturer's described protocols. Briefly, Aβ_1–42 _was dissolved in 0.15 M sodium chloride and Cy3 mono-reactive NHS ester (Amersham Biosciences, Piscataway, NJ) was diluted in dimethyl sulfoxide (DMSO) to a working concentration of 10 mg/mL and this was slowly added to the Aβ_1–42 _solution while stirring. The Cy3-Aβ_1–42 _solution was protected from light while stirred for 45 min at room temperature. To separate the free Cy3-dye, the solution was dialyzed against 1 L of 0.15 M sodium chloride for 4 hr at room temperature. The solution was then exchanged with fresh 0.15 M sodium chloride and dialyzed overnight at 4°C. The next day the Cy3-Aβ_1–42 _solution was dialyzed against 1 L of 0.1 M PBS for 4 hr at room temperature, and again dialyzed overnight using fresh 0.1 M PBS. The solution was then syringe filter sterilized through a 0.22-μm filter and the eluate was aliquoted and stored at -20°C until used. Non-selective cannabinoid agonist (CP 55,940), CB_2 _agonist (JWH-015), and THC were obtained from Tocris (Ellisville, MO) and dissolved in 1% DMSO to a stock concentration of 50 mM.

### Animals and microglial cell cultures

Breeding pairs of BALB/c mice were purchased from Jackson Laboratory (Bar Harbor, ME) and housed in the animal facility at the University of South Florida, College of Medicine. Murine primary culture microglial cells were isolated from mouse cerebral cortices and grown in RPMI 1640 medium supplemented with 5% fetal calf serum (FCS), 2 mM glutamine, 100 U/ml penicillin, 0.1 μg/ml streptomycin, and 0.05 mM 2-mercaptoethanol according to previously described methods [27]. Briefly, cerebral cortices from newborn mice (1–2 day-old) were isolated under sterile conditions and were kept at 4°C before mechanical dissociation. Cells were plated in 75-cm^2 ^flasks and complete medium was added. Primary cultures were kept for 14 days so that only glial cells remained and microglial cells were isolated by shaking flasks at 200 rpm in a Lab-Line incubator-shaker. More than 98% of these glial cells stained positive for microglial marker Mac-1 (CD11b/CD18; Boehringer Mannheim, Indianapolis, IN; data not shown). All animal protocols were approved by the Committee of Animal Research at the University of South Florida, in accordance with the National Institutes of Health guidelines. N9 microglial cells were cultured as previously described [28].

### Reverse transcriptase (RT)-PCR analysis

Total RNA was isolated from primary cultured microglial cells using Trizol reagent (Invitrogen, Carlsbad, CA) as recommended in the manufacturer's protocol. RNA concentration was measured by spectrophotometry at 260 nm. RT-PCR was performed as described previously [28]. Briefly, cDNA was prepared by mixing 1 μg of total RNA from each treatment with an oligo (dT) primer and the MMLV reverse transcriptase (Invitrogen); the reaction mix was incubated in a 37°C water-bath for 50 min before heat inactivation of the mix by increasing the temperature to 70°C for 10 min. This cDNA reaction mixture (20 μl) was diluted with 180 μl of DNAase/RNAase-free water and 10 μL of the cDNA solution was used for gene specific PCR. The PCR primers used were CB_2 _sense: 5'-CCG GAA AAG AGG ATG GCA ATG AAT-3' and antisense: 5'-CTG CTG AGC GCC CTG GAG AAC-3' oligonucleotides were designed to produce the partial 239 bp mouse CB_2 _cDNA (MGI:104650); mouse β-actin sense: 5'-TTG AGA CCT TCA ACA CCC-3' and β-actin antisense: 5'-GCA GCT CAT AGC TCT TCT-3', which yields the 357 bp β-actin cDNA fragment. Samples not undergoing reverse transcription were run in parallel to control for technical errors leading to DNA contamination (data not shown). Mouse β-actin was amplified from all samples as a housekeeping gene to normalize expression. A control (no template) was included for each primer set. PCR was performed with each cycle consisting of 94°C for 1 min, 55°C for 2 min, and 72°C for 2 min, followed by a final extension step at 72°C for 10 min. PCR cycle numbers were kept low to perform semi-quantitative PCR (actin, 25 cycles; CB_2 _30 cycles). PCR products were resolved on 1.2% ethidium bromide-stained agarose gels, and visualized by ultraviolet transillumination.

### Flow cytometric analysis of microglial CD40 expression

Primary cultured microglial cells were plated in 6-well tissue culture plates at 5 × 10^5 ^cells/well and incubated with THC, CP55940 or CB_2 _agonist (JWH-015) at different doses in the presence or absence of IFN-γ (100 U/ml). Twelve hours after incubation, these microglial cells were washed with flow buffer [PBS containing 0.1% (w/v) sodium azide and 2% (v/v) FCS] and re-suspended in 250 μl of ice-cold flow buffer for fluorescence activated cell sorting (FACS) analysis, according to methods described previously [28]. Briefly, cells were pre-incubated with anti-mouse CD16/CD32 monoclonal antibody (clone 2.4G2, PharMingen, Los Angeles, CA) for 10 min at 4°C to block non-specific binding to Fc receptors. Cells were then spun down at 5,000 g washed 3 times with flow buffer and then incubated with hamster anti-mouse CD40-FITC or isotype control antibody-FITC (1:100 dilution; PharMingen) in flow buffer. After 30 min incubation at room temperature, cells were washed twice with flow buffer, re-suspended in 250 μL of flow buffer and analyzed by a FACScan™ instrument (Becton Dickinson, Franklin Lanes, NJ). A minimum of 10,000 cells were accepted for FACS analysis. Cells were gated based on morphological characteristics such that apoptotic and necrotic cells were not accepted for FACS analysis using CellQuest™ software (Beckton Dickinson). Percentages of positive cells (i.e. CD40-expressing) were calculated as follows: for each treatment, the mean fluorescence value for the isotype-matched control antibody was subtracted from the mean fluorescence value for the CD40-specific antibody.

### Western immunoblotting analysis

Murine microglial cell lysates (including primary cultured microglial cells) were prepared in ice-cold lysis buffer (20 mM Tris, pH 7.5,150 mM NaCl, 1 mM EDTA, 1 mM EGTA, 1% Triton X-100, 2.5 mM sodium pyrophosphate, 1 mM glycerolphosphate, 1 mM Na_3_VO_4_, 1 μg/ml leupeptin, and 1 mM PMSF) and protein concentration was determined by the Bio-Rad protein assay as previously described [29]. An aliquot corresponding to 100 μg of total protein of each sample was separated by SDS-PAGE and transferred electrophoretically to immunoblotting PVDF membranes. Nonspecific antibody binding was blocked with 5% nonfat dry milk for 1 hr at room temperature in Tris-buffered saline (20 mM Tris and 500 mM NaCl, pH 7.5). Subsequently, these membranes were first hybridized with the goat anti-CB_2 _antibody (1:100 dilution; Santa Cruz) for 2 hr and then washed 3 times in TBS and immunoblotting using an anti-goat HRP-conjugated IgG secondary antibody as a tracer (Pierce Biotechnology, Inc. Rockford, Illinois). Luminol reagent (Pierce Biotechnology, Inc.) was used to develop the blots. To demonstrate equal loading, the same-membranes were then stripped with β-mercaptoethanol stripping solution (62.5 mM Tris-HCl, pH 6.8,2% SDS, and 100 mM β-mercaptoethanol), and finally re-probed with mouse monoclonal antibody to β-actin (Pierce Biotechnology, Inc.).

### Immunochemistry analysis

Six mice (10 weeks of age, 3 male/3 female, C57 BL/6N; Crea, Tokyo, Japan) were used to examine the expression of CB_2 _in microglial cells. After mice were euthanized with an overdose of sodium pentobarbital (50 mg/kg), the brain was perfused transcardinally with 200 mL of 10 U/mL heparin in saline followed by 200 mL of 4% paraformaldehyde in 0.1 M (pH 7.4) PBS. The brains were removed and fixed in the same fixative overnight at 4°C, dehydrated, and routinely embedded in paraffin with 16 hr processing. For *in situ *detection of CB_2_, sections (5 μm in thickness) were deparaffinized and pretreated by hydrolytic autoclaving in 10 mM citrate buffer (pH 6.0) for 15 min at 121°C to retrieve antigens. Thereafter, sections were treated with endogenous peroxidase quenching (0.3% H_2_O_2 _for 10 min) and pre-blocked with serum-free blocking solution (DAKO, Carpinteria, CA) for 30 min prior to primary antibody incubation. Immunohistochemistry was performed according to the manufacturer's protocol using the Vectastain ABC Elite kit (Vector Laboratories, Burlingame, CA) coupled with the diaminobenzidine reaction. For double labeling of CB_2 _and Iba-1 (microglial cell marker) in frozen sections, an additional six mice were euthanized with the same anesthesia as above, and then the brains were perfused transcardially with 200 mL of 10 U/mL heparin in saline. Brains were quick-frozen at -80°C for cryo-sectioning (5 μm in thickness). Prior to immunohistochemistry, frozen sections were fixed with 4% paraformaldehyde in 0.1 M (pH 7.4) PBS for 1 hr, and pre-blocked with serum-free blocking solution (DAKO, Carpinteria, CA) for 30 min. The following primary and secondary antibodies were used: goat anti-mouse CB_2 _antibody (1:400 dilution; Santa Cruz Biotechnologies), rabbit anti-C-terminus of Iba-1 antibody (1:500 dilution; Wako Pure chemical Industries, Osaka, Japan), FITC-conjugated donkey anti-goat IgG (1:50 dilution; Jackson ImmunoResearch Laboratories, West Grove, PA), and TRITC-conjugated swine anti-rabbit IgG (1:50 dilution; DAKO, Carpinteria, CA). In addition, for a neutralization test (pre-absorption test), Goat anti-mouse CB_2 _antibody was pre-incubated for 30 min with a five-fold (w/v) excess of mouse CB_2 _blocking peptides (Santa Cruz Biotechnologies). Whereas the appropriate isotype control serum or PBS was used instead of primary antibody or ABC reagent as a negative control, spleen was used as a positive control. Counterstaining was performed with hematoxylin.

### CB_2 _small interfering RNA

N9 cells were transfected with specific murine CB_2 _targeting siRNA designed to knockdown murine CB_2 _expression (Humesis Biotechnology Corporation, New Orleans, LA). Briefly, N9 cells were seeded in 24-well plates and cultured until they reached 70% confluency. The cells were then transfected with 100 nM anti-CB_2 _siRNA or anti-green fluorescent protein (GFP; non-targeting control; Humesis) using Code-Breaker transfection reagent (Promega, Madison, WI) and cultured for an additional 18 hr in serum-free MEM. The cells were allowed to recover for 24 hr in complete medium (MEM 10% FBS) before treatments. The cells were evaluated by Western immunodetection for the expression of CB_2 _using anti-CB_2 _antibodies (Santa Cruz) following siRNA treatment. The cells were also cultured for 4 hr with LPS, JWH-015, or various combinations, and TNF-α release was measured by specific enzyme-linked immunosorbent assay (ELISA). Transfection efficiency was determined to be greater than 80% (data not shown) using no-RISC siGLOW obtained from Dharmacon (Lafayette, CO).

### TNF-α and NO (nitric oxide) analyses

Murine primary cultured microglial cells were plated in 24-well tissue-culture plates (Costar, Cambridge, MA) at 1 × 10^5 ^cells per well and stimulated for 24 hr with either IFN-γ (100 U/ml)/CD40L protein (2.5 μg/ml) or Aβ_1–42 _(3 μM)/CD40L protein (2 μg/ml) in the presence or absence of CB_2 _agonist JWH-015 (5 μM). Cell-free supernatants were collected and stored at -70°C until analysis. TNF-α and NO levels in the supernatants were examined using ELISA kits (R&D Systems) and NO assay (Calbiochem) in strict accordance with the manufacturers' protocols. Cell lysates were also prepared and the Bio-Rad protein assay (Hercules, CA) was performed to measure total cellular protein. Results are shown as mean pg of TNF-α or NO per mg of total cellular protein (+/- SD).

### JAK/STAT1 signaling pathway analysis

Primary culture microglial cells were plated in 6-well tissue culture plates at a density of 5 × 105 cells per well and co-incubated with IFN-γ (100 U/mL) in the presence or absence of a dose range of CB_2 _agonist (0.31, 0.62, 1.25, 2.5 and 5.0 μM) for 30 min. At the end of the treatment period, microglial cells were washed in ice-cold PBS three times and lysed in ice-cold lysis buffer. After incubation for 30 min on ice, samples were centrifuged at high speed for 15 min, and supernatants were collected. Total protein content was estimated using the Bio-Rad protein assay. For phosphorylation of JAK1 and JAK2, membranes were first hybridized with phospho-specific Tyr1022/1023 JAK1 or Tyr1007/1008 JAK2 antibody (Cell Signaling Technology, Beverly, MA) and then stripped and finally analyzed by total JAK1 or JAK2 antibody. For STAT1 phosphorylation, membranes were probed with a phospho-Ser727 STAT1 antibody (Cell Signaling Technology) and stripped with stripping solution and then re-probed with an antibody that recognizes total STAT1 (Cell Signaling Technology). Alternatively, membranes with identical samples were probed either with phospho-JAK or STAT1, or with an antibody that recognizes total JAK or STAT1. Immunoblotting was performed with a primary antibody followed by an anti-rabbit HRP-conjugated IgG secondary antibody as a tracer. After washing in TBS the membranes were incubated in luminol reagent and exposed to x-ray film.

### Microglial Aβ phagocytosis assays

Microglial phagocytosis of fibrillar/aggregated Aβ_1–42 _peptide was carried out in a manner similar to previously described protocols [30-32]. Microglial cells were cultured at 5 × 10^5^/well in 6-well tissue-culture plates with glass inserts (for fluorescence microscopy). The following day, microglial cells were treated with Cy3-conjugated Aβ_1–42 _(3 μM) and CD40L protein (2.5 μg/mL) in the presence or absence of CB_2 _agonist (5 μM) for 3 hr. In parallel dishes, microglial cells were incubated with Cy3-conjugated Aβ_1–42 _under the same treatment conditions above except they were incubated at 4°C to control for non-specifically cellular association of Cy3-Aβ_1–42. _Microglial cells were then rinsed 3 times in Aβ_1–42_-free complete medium and the medium was exchanged with fresh Aβ_1–42_-free complete medium for 10 min both to allow for removal of non-incorporated Cy3-Aβ_1–42 _and to promote concentration of the Cy3-Aβ_1–42 _peptide into phagosomes. This medium was withdrawn and microglial cells were rinsed 3 times with ice-cold PBS. For fluorescence microscopy, microglial cells on glass coverslips were fixed for 10 min at 4°C with 4% (w/v) paraformaldehyde (PFA) diluted in PBS. After three successive rinses in TBS, microglial cell nuclei were detected by incubation with DAPI for 10 min and finally mounted with fluorescence mounting media containing Slow Fade antifading reagent (Molecular Probes, Eugene, OR) and then viewed under an Olympus IX71/IX51 fluorescence microscope equipped with a digital camera system to allow for digital capture of images (40×).

For immunoblot detection of cell-associated Aβ, primary microglial cells were plated in 6-well tissue culture plates with glass inserts at 5 × 10^5 ^cells/well and treated as described for immunofluorescense detection of Cy3-Aβ_1–42 _except that these experiments employed Aβ_1–42_. Immunoblotting was carried out with the monoclonal anti-human Aβ antibody (BAM-10, 1:1,000 dilution; Sigma) followed by an anti-mouse IgG-HRP as a tracer. Blots were developed using the Immun-Star chemiluminescence substrate. The membranes were stripped and then re-probed with a reference anti-mouse β-actin monoclonal antibody, which allows for quantification of the band density ratio of Aβ to β-actin by densitometric analysis.

### Statistical analysis

Data are presented as mean +/- SD. All statistics were analyzed using a one-way multiple-range analysis of variance test (ANOVA) for multiple comparisons. A value of p < 0.05 was considered significant.

## Results

### Stimulation of CB_2 _inhibits IFN-γ-induced CD40 expression in microglial cells

**Figure 1:**
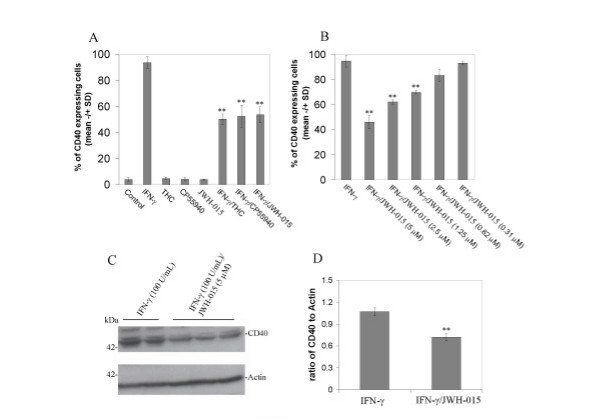
**Cannabinoids inhibit microglial CD40 expression induced by IFN-γ**. A, Mouse primary microglial cells were cultured in 6-well tissue-culture plates (5 × 10^5^/well) and treated with THC (0.6 μM), CP55940 (5 μM) or selective cannabinoid CB_2 _agonist (JWH015; 5 μM) in the presence or absence of IFN-γ (100 U/mL), or treated with vehicle (1% DMSO Control) or IFN-γ alone (100 U/mL); B, In parallel 6-well tissue-culture plates, microglial cells were incubated with IFN-γ (100 U/mL) in the presence or absence of JWH-015 at the indicated doses. After 12 hr-treatments, these cells were prepared for FACS analysis of CD40 expression as described in Materials and methods. For A, ANOVA and *post hoc *testing showed significant differences of mean fluorescence (+/- SD with n = 3 for each condition) between IFN-γ treatment and IFN-γ treatment in the presence of THC, CP55940 or JWH-015 (p < 0.001). However, there was not a significant difference between IFN-γ/THC and either IFN-γ/CP55940 or IFN-γ/JWH-015 (p > 0.05). For B, ANOVA and *post hoc *testing showed significant differences of mean fluorescence (+/- SD with n = 3 for each condition) between IFN-γ treatment and IFN-γ treatment in the presence of JWH-015 at 5 μM, 2.5 μM and 1.25 μM (** p < 0.001). C, Western blot analysis by anti-mouse CD40 antibody shows CD40 protein expression and, by anti-β-actin antibody, shows β-actin protein (internal reference). D, Densitometric quantification of Western immunoblotting analysis from independent experiments (n = 2 for IFN-γ; n = 3 for IFN-γ/JWH-015 treatment) indicated that doses of JWH-015 of 1.25 μM or greater significantly (** p < 0.05) reduced IFN-γ-induced CD40 expression. CD40 expression is shown normalized to β-actin.

In previous studies, we and others showed that expression of constitutive levels of CD40 on microglial cells can be induced in response to IFN-γ challenge [28,33]. We recently reported that lovastatin treatment inhibits CD40 expression in cultured microglial cells [34]. To investigate cannabinoid regulation of CD40 expression in microglial cells, primary cultured murine microglial cells were treated with IFN-γ (100 U/ml) in the presence or absence of THC, CP55940 or JWH-015 for 12 hr and the expression of CD40 was analyzed by flow cytometry. As expected, the treatment of cultured microglial cells with THC, CP55940 and JWH-015 significantly inhibited CD40 expression induced by IFN-γ (Figure 1A). Treatment with the CB_2 _agonist, JWH-015, inhibited IFN-γ-induced CD40 expression in a dose-related manner (Figure. 1B). Furthermore, Western blotting examination consistently showed that JWH-015 co-treatment mitigates the inducible increase in CD40 protein expression in primary cultured microglial cells after IFN-γ treatment (Figure. 1C, D). Taken together, these findings suggest that stimulation of CB_2 _decreases CD40 expression on primary cultured microglial cells.

### Microglial cells express CB_2_

**Figure 2:**
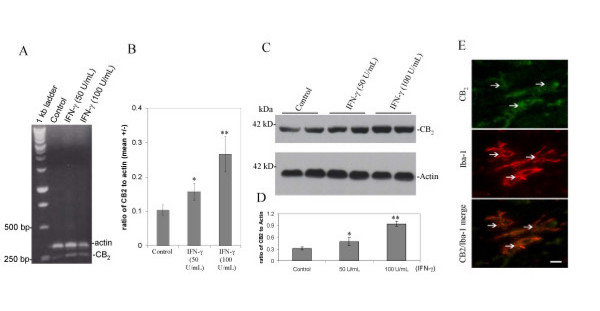
**Cannabinoid receptor CB_2 _is expressed by cultured microglial cells**. A, RT-PCR analysis of murine primary cultured microglial cells. A 239-bp band corresponding to CB_2 _was specifically generated with primers described in the Materials and methods section. B, Graphical representation of RT-PCR band density ratio of CB_2 _expression normalized to β-actin (mean +/- SD) is shown (n = 3 for each condition). ANOVA revealed significant between-group differences (control *versus *IFN-γ (50 U/mL) and IFN-γ (50 U/mL) *versus *IFN-γ (100 U/mL); p < 0.005). C, Western immunoblot analysis of murine primary cultured microglial cells using specific antibodies targeting CB_2 _and β-actin proteins. D, Western blot band density is represented as ratio of CB_2 _to β-actin (mean +/- SD; n = 4 for each condition). ANOVA revealed significant between-group differences [Control *versus *IFN-γ (50 U/mL) and IFN-γ (50 U/mL) *versus *IFN-γ (100 U/mL); ** p < 0.005]. E, Cannabinoid receptor CB_2 _is expressed in microglial cells *in situ*. In white matter, microglial cells are positive in their somata and processes for CB_2_. White arrowheads show positive cells as indicated. The expression of CB_2 _(FITC; green) was co-localized with Iba-1, microglial cell marker (TRITC; red) as indicated. Bottom panel denotes merge signals. Bar denotes 10 μm.

In order examine whether CB_2 _might be expressed in cultured microglial cells, we first isolated total RNA from primary cultured microglial cells for reverse transcriptase-polymerase chain reaction (RT-PCR) analysis. Results show that CB_2 _mRNA is constitutively expressed in primary cultured microglial cells (Figure. 2A) and, more importantly, is significantly increased following IFN-γ(50 U/ml and 100 U/ml) challenge (Figure. 2A, B). Furthermore, Figure 2C and 2D, show that CB_2 _protein is detected in primary cultured microglial cells, and is also markedly increased following the challenge with IFN-γ, by Western blotting. To further evaluate CB_2 _expression in microglial cells, we performed immunohistochemistry on adult mouse brain, and found that adult mouse microglial cells stained positively for CB_2 _(Figure. 2E, top). To rule out the possibility that microglial cells non-specifically bound anti-CB_2 _antibody, we pre-absorbed the goat anti-mouse CB_2 _antibody with mouse CB_2 _blocking peptide. The CB_2 _signal is markedly reduced in mouse brain when the blocking peptide is employed (data not shown). Moreover, immunohistochemical analysis indicated that expression of CB_2 _by microglial cells was co-localized with microglial cell marker Iba-1 (Figure. 2E, bottom).

### Anti-CB_2 _small interfering RNA blocked effect of CB_2 _agonist JWH-015 treatment

**Figure 3:**
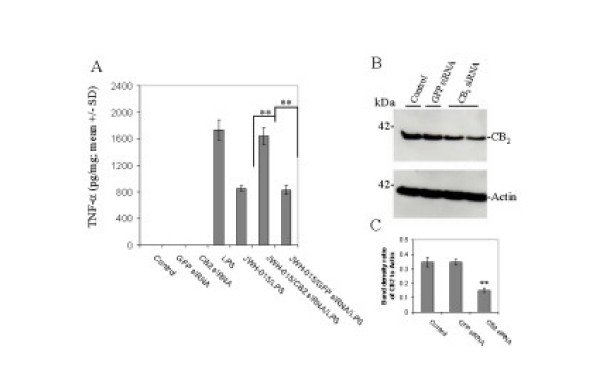
**Cultured microglial cells (N9) treated with LPS and 100 nM anti-murine CB_2 _siRNA lose their ability to respond to CB_2 _agonist, JWH-015**. A, Microglial cells treated with LPS (100 ng/mL) secreted large quantities of TNF-α (n = 3, **p < 0.005). Co-treatment with JWH-015 (5 μM) attenuated LPS-induced TNF-α release. Pre-treatment with anti-CB_2 _siRNA abolished JWH-015's ability to reduce LPS-induced TNF-α release (n = 3, ** p < 0.05). Non-targeting anti-GFP siRNA control had no effect. B and C, Western blot using an anti-murine CB_2 _antibody demonstrates that 100 nM anti-CB_2 _siRNA significantly reduced expression of CB_2 _protein by N9 microglial cells after 48 hr (n = 2, ** p < 0.05).

N9 cells, transfected for 18 hr with specific murine CB_2 _targeting siRNA (100 nM), were treated for 4 hr with LPS, JWH-015, or in various combinations, and TNF-α release was measured by ELISA (Figure 3A). Anti-CB_2 _siRNA was able to completely abolish JWH-015-mediated reductions in LPS-induced TNF-α release. In addition, to evaluate the knock-down efficiency, we performed Western blot using anti-CB_2 _antibody and found a significantly decreased level of CB_2 _expression in siRNA transfected condition (Figure 3B). These data indicate that JWH-015 is activating CB_2 _to oppose the TNF-α release caused by LPS treatment.

### CB_2 _agonist inhibited JAK/STAT signaling induced by IFN-γ in microglial cells

**Figure 4:**
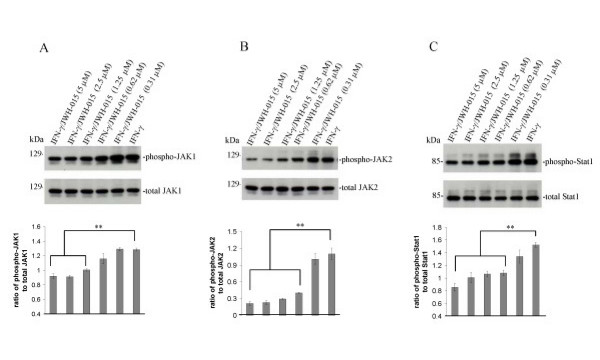
**Cannabinoid CB_2 _agonist treatment opposes IFN-γ-induced phosphorylation of JAK/STAT1 in microglial cells**. A, B, Primary microglial cells were seeded in 6-well tissue-culture plates (5 × 10^5^/well) and treated with IFN-γ (100 U/mL) in the presence or absence of CB_2 _agonist (JWH-015) at the indicated doses for 30 min. Cell lysates were prepared from these cells and subjected to Western immunoblotting using antibodies against phospho-JAK1 (Tyr1022/1023) and JAK2 (Tyr1007/1008), or total JAK1 and JAK2, as indicated. Densitometric quantification of all Western immunoblots results are summarized by the histograms below, representative of Western immunoblots from two independent experiments. Dose-dependent reductions in phospho-JAK1/total JAK1 and phosphor-JAK2/total JAK2 correlated with JWH-015 treatments, becoming significant (** p < 0.05) at doses greater than or equal to 1.25 μM and 0.62 μM for JAK1 and JAK2, respectively. C, In parallel experiments, cell lysates were subjected to Western immunoblotting using anti-phospho-STAT1 (Ser727) or anti-total STAT1 antibody as indicated. Dose-dependent reductions in phospho-Stat1/total Stat1 correlated with JWH-015 treatments, becoming significant (** p < 0.05) at doses greater than or equal to 0.62 μM.

Previous reports demonstrate the ability of IFN-γ to potently induce microglial CD40 expression [28]. The signal transduction pathway involved in this induction most likely involves elements of the JAK/STAT signaling pathway [35,36]. Interestingly, many of the factors (cytokines, neurotrophins, neuropeptides, statins) that inhibit IFN-γ-induced microglial CD40 expression do so by modification of the JAK/STAT pathway [34-39]. Therefore, we examined the effects of stimulation of CB_2 _on the JAK/STAT signaling pathway in primary cultured microglial cells. Cultured microglial cells were treated with IFN-γ for 30 min in the presence or absence of a dose range of CB_2 _agonist JWH-015. Western immunoblotting analysis revealed that JWH-015 treatment markedly mitigated JAK1 Tyr1022/1023 and JAK2 Tyr1007/1008 phosphorylation in dose-dependent manner (Figure. 4A, B). Further, it is well known that during IFN-γ interaction with its heterodimer type II cytokine receptor, the JAKs are directly activated leading to STAT1 phosphorylation [35,36,38]. Accordingly, we examined the effects of CB_2 _stimulation on STAT1 phosphorylation, in the same dose range mentioned above, on primary microglial cells treated with IFN-γ for 30 min. Results showed that JWH-015 co-treatment significantly inhibited Ser727 phosphorylation of the STAT1 protein at 10 μM (Figure. 3C). Unstimulated microglial cells displayed very little detectable JAK1,2 or STAT-1 phosphorylation (data not shown).

### Stimulation of CB_2 _inhibits functional CD40 signaling in microglial cells

**Figure 5:**
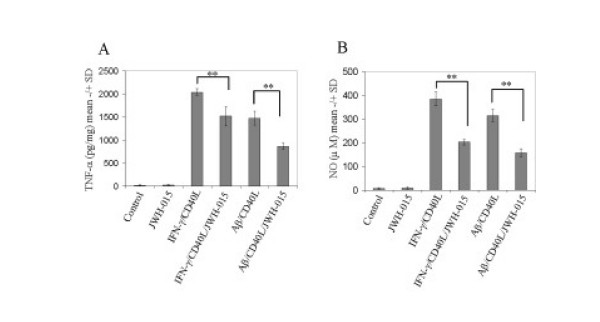
**CB_2 _stimulation attenuates microglial proinflammatory cytokine release**. Mouse primary microglial cells were seeded in 24-well tissue-culture plates (1 × 10^5^/well) and co-treated with either IFN-γ (100 U/mL)/CD40L protein (2 μg/mL) or Aβ_1–42 _(1 μM)/CD40L protein (2 μg/mL) in the presence or absence of cannabinoid receptor CB_2 _agonist (JWH015, 5 μM) for 24 hr. Cell cultured supernatants were collected and subjected to TNF-α cytokine ELISA (A) and NO release assay (B) as indicated. TNF-α production was represented as mean pg of TNF-α per mg of total cellular protein (+/- SD). Similar results were obtained in three independent experiments. ANOVA and *post hoc *testing revealed significant differences between IFN-γ/CD40L and IFN-γ/CD40L and JWH-015 (** p < 0.005); Aβ_1–42_/CD40L and Aβ_1–42_/CD40L plus JWH-015 treatment (** p < 0.001).

**Figure 6:**
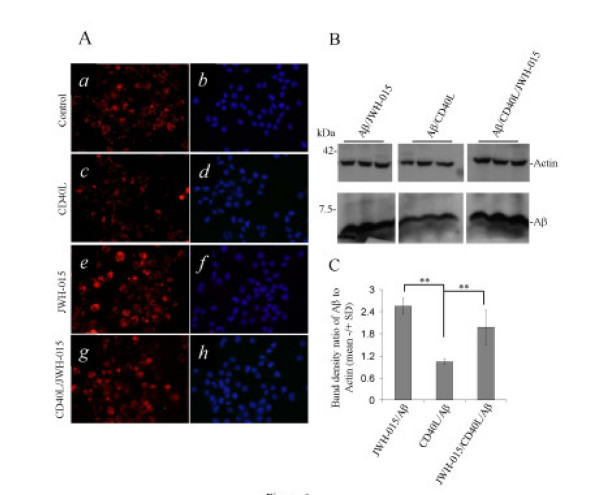
**CB_2 _stimulation modulates microglial phagocytic function**. A, Mouse primary microglial cells were seeded in 6-well tissue culture plates with glass inserts (5 × 10^5^cells/well) and treated with 3 μM Cy3™-Aβ_1–42 _in the absence (*a *and *b*; Control) or presence of either CD40L protein (*c *and *d *2.5 μg/mL) or JWH-015 (*e *and *f*; 5 μM), or both JWH-015 and CD40L protein (*g *and *h*). After 3 hr these cells were washed and fixed (see Materials and Methods). Subsequently, immunofluorescence microscopy examination was performed using a 40 X objective with appropriate filter selection. The darkfield images a, c, e, and g show the fluorescence of Cy3™ labeled Aβ_1–42 _whereas, b, d, f, and h show only the DAPI nuclear stain of the same fields. B, In parallel experiments, under the same treatment conditions, microglial cell lysates were prepared for Western immunoblotting analysis (see Materials and methods) of cell-associated Aβ_1–42 _using anti-Aβ antibody (BAM-10, Sigma). C, Aβ mean band densities are graphically represented as ratios to β-actin +/- SD (n = 3 for each condition). ANOVA revealed significant between-group differences (JWH-015/Aβ *versus *CD40L/Aβ and Aβ/CD40L *versus *JWH-015/CD40L/Aβ; ** p < 0.005), and *post hoc *testing showed significant differences between CD40L/Aβ and JWH-015/CD40L/Aβ (** p < 0.005).

To examine the functional consequences of CB_2 _agonist treatment on CD40 expression, we stimulated mouse primary microglial cells with either IFN-γ/CD40L protein [28,40,41] or Aβ_1–42_/CD40L protein in the presence or absence of JWH-015 for 24 hr. Supernatants from each treatment condition were examined by ELISA for pro-inflammatory molecules that we have previously described as being induced by microglial CD40 ligation [14,27-31]. As we expected, ELISA measurements revealed that either IFN-γ /CD40L or Aβ_1–42_/CD40L increased the secretion of the pro-inflammatory molecules TNF-α and NO, as indicated in Figure 5A and 5B. However, when CB_2 _is stimulated by the presence of JWH-015, these pro-inflammatory molecules were significantly reduced. The canonical microglial function in the CNS is thought to be phagocytosis, and given that IFN-γ and CD40 signaling are maturation agents that oppose this phagocytic function [15,42-47], we examined whether CB_2 _agonist co-treatment could rescue microglial phagocytic function. Murine primary microglial cultures were exposed to 3 μM of Aβ_1–42 _(for immunoblotting) or Cy3™-Aβ_1–42 _(for phagocytosis assay) in the presence or absence of CD40L protein or CD40L protein/JWH-015. After 3 hr, the amount of phagocytosed Aβ_1–42 _peptide was determined by both qualitative immunofluorescence studies (Figure 6A) and with quantitative immunoblotting experiments (Figure 6B and 6C). As shown in Figure 6A, CD40 ligation decreased microglial phagocytic function compared to controls (Figure 6A, panel *a*, *b *versus *c*, *d*), while CB_2 _agonist treatment alone increased compared to control (Figure 6A, panel *a*, *b *versus *c*, *d*). Interestingly, the presence of JWH-015 rescued microglial phagocytosis of Cy3-Aβ_1–42 _following CD40L treatment (Figure. 6A, panel *g*, *h *versus *e*, *f*). In a parallel experiment, we further showed that CB_2 _stimulation by JWH-015 resulted in a significant attenuation of CD40L-mediated impairment of microglial phagocytosis of Aβ_1–42_, as evidenced by increased band density ratio of Aβ to β-actin using Western immunoblotting (Figure. 6B and 6C).

## Discussion

The findings of the present study suggest that cannabinoids, namely CB_2 _agonist JWH-015, reduce IFN-γ-induced up-regulation of CD40 expression in mouse microglial cells by interfering with the JAK/STAT1 pathway. Given that this finding is consistent with the immunosuppressive effects of cannabinoids reported previously [48], the significance of our present findings must be considered in the context of the function of microglial CD40.

Aberrant expression of CD40 by microglial cells, in conjunction with the release of TNF-α, is directly correlated with pathogenic events occurring in the CNS of MS patients [49-51] and in the EAE mouse model of MS [52]. In AD, activated microglial cells are considered a major contributor to the local inflammatory responses evidenced in neuritic plaques. Furthermore, the CD40-CD40L dyad is potentiated, as can be seen from the increased numbers of CD40-positive microglial cells as well as increased CD40L expression on astrocytes in AD [53,54]. Our previous work has shown a correlation between increased levels of Aβ peptide and enhanced CD40 expression on microglial cells derived from the Tg2576 mouse model of AD [28]. We also reported that Aβ peptide can synergize with the IFN-γ signaling pathway to induce microglial CD40 expression and subsequent neurotoxicity [28].

A review of the molecular basis of CD40 expression in macrophages/microglial cells illuminates the critical role of the JAK/STAT1 pathway [55]. In this study, we show that the CB_2 _agonist JWH015 inhibits IFN-γ-induced microglial CD40 expression by opposing JAK/STAT1 pathway activation. One possible mechanism of JWH015's inhibition of the JAK/STAT1 pathway is provided by a recent report showing that treatment with novel cannabinoid, PRS-211,092, significantly decreased Concanavalin A-induced liver injury in mice that was accompanied by an induction of early gene expression of the suppressors of cytokine signaling (SOCS-1 and 3). The SOCS proteins act as negative regulators of the JAK/STAT1 pathway either by binding and inhibiting JAK tyrosine kinases or by inhibiting binding of STAT1 factors to the cytoplasmic domains of the receptors [56].

We previously reported that mechanisms that antagonize microglial CD40 expression or CD40 signaling could also block microglial production of proinflammatory mediators [27]. In this study, we have also shown that CB_2 _agonist JWH-015 similarly inhibits microglial CD40 ligation-induced production of proinflammatory cytokines. This finding is consistent with studies showing that CB_2 _agonists inhibit microglial production of proinflammatory mediators [22]. These data, suggesting that the CB_2 _agonist JWH-015 promotes microglial phagocytic function, are of great interest given that mechanisms driving the clearance of cerebral Aβ underlie principles of many therapeutic strategies for AD.

## List of abbreviations

Aβ : Amyloid-β peptide

CD40: CD40 receptor

CD40L: CD40 ligand

CNS: Central nervous system

HIV: Human immunodeficiency virus

IFN-γ : Interferon-gamma

JAK: Janus kinase

MHC II: Major histocompatibility complex II

STAT1: Signal transducer and activator of transcription 1

TNF-α : Tumor necrosis factor-alpha

## Competing interests

The author(s) declare that they have no completing interests.

## Authors' contributions

JE carried out flow cytometric analysis, RT-PCR, TNF-a/NO analysis, experimental analysis and data interpretation, and prepared the manuscript. DO performed the CB_2 _small interfering RNA assays and aided in the preparation of the manuscript. TM performed the CB_2 _immunohistochemistry analysis. HH and BY performed microglial Aβ phagocytosis assays. NS carried out Western blots for JAK/STAT1 signaling pathway analysis. TK, FF, JT and RDS conceived the design of the study, aided in the preparation of the manuscript, and provided critical analysis of the manuscript.
